# Four-Week Evaluation of the Interaction Pattern Among Saccharibacteria, Nitrate-Reducing Bacteria, and Periodontopathogens in Orthodontic Miniscrew Implants

**DOI:** 10.3390/dj13090405

**Published:** 2025-09-04

**Authors:** Boy M. Bachtiar, Endang W. Bachtiar, Nicholas S. Jakubovics, Turmidzi Fath, Sariesendy Sumardi, Nada Ismah, Natalina Haerani, Fatimah Maria Tadjoedin, Zamri Radzi

**Affiliations:** 1 Department of Oral Biology, Faculty of Dentistry, Universitas Indonesia, Jakarta 10430, Indonesia; boy_mb@ui.ac.id (B.M.B.); turmidzi.fath02@ui.ac.id (T.F.); 2 Oral Science Research Center, Faculty of Dentistry, Universitas Indonesia, Jakarta 10430, Indonesia; 3 Centre for Oral Health Research, School of Dental Sciences, Faculty of Medical Sciences, Newcastle University, Framlington Place, Newcastle upon Tyne NE2 4BW, UK; nick.jakubovics@newcastle.ac.uk; 4 Deparment of Orthodontics, Faculty of Dentistry, Universitas Indonesia, Jakarta 10430, Indonesia; sariesendy.sumardi@ui.ac.id (S.S.); nada.ismah@ui.ac.id (N.I.); 5 Department of Periodontics, Faculty of Dentistry, Universitas Indonesia, Jakarta 10430, Indonesia; natalina_perio@ui.ac.id (N.H.); fatimah.tadjoedin@ui.ac.id (F.M.T.); 6 Department of Pediatric Dentistry & Orthodontics, Faculty of Dentistry, University of Malaya, Kuala Lumpur 98115, Malaysia; zamrir@um.edu.my

**Keywords:** orthodontics, mini-implants, oral microbiome, Saccharibacteria, periodontopathogens, nitrate-reducing bacteria

## Abstract

**Background/Objective**: Orthodontic mini-implants (MI) create new niches that may alter the oral microbiota and modulate host immune responses. While clinical inflammation is not always evident, microbial and molecular changes may precede visible signs of peri-implant infection. This study investigated microbial shifts and inflammatory responses following MI placement, with a focus on Saccharibacteria, nitrate-reducing bacteria (NRB), and periodontopathogens. **Methods**: Saliva and peri mini-implant crevicular fluid (PMICF) samples were collected from eight orthodontic patients at baseline (T0), one week (T1), and one month (T2) after mini-implant placement. DNA was extracted from each saliva and PMICF sample and pooled across the eight patients for each time point. The pooled DNA were then subjected to 16S rRNA gene sequencing using the Oxford Nanopore MinION platform. Statistical analysis was performed to determine shifts in bacterial abundance, diversity, and co-occurrence patterns across the different sample types (saliva vs. PMICF) and time points. **Results**: Alpha diversity decreased in PMICF at T2, while it remained stable in saliva samples. Periodontopathogens (*Porphyromonas gingivalis*, *Treponema denticola*, *Fusobacterium nucleatum*) increased in PMICF at T2, while NRB and Saccharibacteria, along with a representative host bacterium (*Schaalia odontolytica*), remained relatively stable. Co-occurrence analysis showed antagonistic relationships between Saccahribacteria/NRB and periodontopathogens. IL-6 significantly decreased from T1 to T2, while CRP showed a non-significant downward trend. The expression of nitrate reductase genes *narG* and *napA* remained stable across time intervals. **Conclusions**: Despite no clinical inflammation, MI placement led to localized microbial shift and mild inflammatory responses. NRB and Saccharibacteria’s stability and antagonistic relationship to periodontopathogens may indicate that they could be involved in maintaining microbial homeostasis. These findings highlight possible early biomarkers and ecological strategies to support oral health in MI patients.

## 1. Introduction

The human oral cavity harbors a complex microbial ecosystem, with distinct habitats supporting unique microbial populations [1]. Given its accessibility, the oral cavity is valuable for studying microbial interactions [2,3]. Mini-implants (MIs) are one type of orthodontic appliance that can change this oral ecosystem [4,5,6]. Placing these in alveolar bone provides new additional spaces for microbial colonization [7,8,9].

One major contributing factor to dental implant failure is peri-implantitis, which is mostly caused by bacterial dysbiosis [10,11]. Thus, it is crucial to understand these microbial changes following MIs implantation since oral host-microbiome balance is essential for sustaining oral health. When MI placement disrupts normal oral homeostasis and function, the ongoing host–microbiome interaction determines whether balance is restored or breakdown results in disease. While some bacteria, such as nitrate-reducing bacteria (NRB) and Saccharibacteria, may be important for health, the presence of specific periodontopathogens in dental or peri-implant plaque is related to disease [12,13,14,15,16].

Saccharibacteria were commonly detected in oral samples by DNA sequencing analysis for many years, but the first report of laboratory culture was not until 2015 [17]. Since then, several different strains of Saccharibacteria have been cultured in the laboratory. All of them are ultrasmall bacteria that require larger bacteria as hosts to grow. So far, the only hosts that have been identified for oral Saccharibacteria are *Actinomycetota* belonging to the genera *Actinomyces*, *Arachnia*, or *Schaalia*. The presence of its host bacterium frequently coincides with the detection of Saccharibacteria (TM7) because it is unable to survive on its own.

In terms of health and illness, the bacterial phylum Saccharibacteria is one of the most challenging in the human oral microbiome [18]. The bacteria are often prevalent in periodontitis [19,20,21,22]. However, it is not clear whether they contribute to the disease process, and there is some evidence that they may protect against periodontal disease in some circumstances [23]. Additionally, certain *Actinobacteria* hosts, such *Schaalia odontolytica* (previously *Actinomyces odontolyticus*) [19], have a symbiotic relationship with Saccharibacteria (TM7). Since *Actinomyces* constitute the majority of bacteria in the microbiomes of healthy individuals [24], interest in the ability of TM7-host interactions in various oral conditions, such as the placement of MIs, is still significant. Apart from TM7, NRB like *Rothia, Neisseria, Haemophilus*, and *Veillonella* are important for maintaining oral and systemic heath because they convert dietary nitrate into nitrite [13] and help preserve oral microbial homeostasis [25]. Dysbiosis, high blood pressure, and other systemic disorders have been associated with decreased nitrate-reducing activity [26]. We reasoned that the microbial profile could alter both temporally and persistently within a person during orthodontic treatment. Therefore, in order to obtain a better understanding of how MI-based orthodontic therapies affect oral health, this study examined changes in the oral microbiota of patients undergoing fixed orthodontic therapies. Specifically, we tracked changes in the microbiota for a month following the placement of MIs. We aimed to assess the relative abundance of periodontopathogens (*Porphyromonas gingivalis*, *Treponema denticola*, *Tannerella forsythia*, and *Fusobacterium nucleatum*), nitrate-reducing bacteria/NRB (*Rothia, Niesseria, Haemophilus, Actinomyces*, and *Veillonella*), and *Saccharibacteria* and its bacterial host (*S. odontolytica*). The minION Nanopore sequencing was employed to provide long-read, cost-effective, and rapid analysis of the bacterial composition [27,28].

## 2. Materials and Methods

### 2.1. Patient Recruitment

This study included eight patients (18–30 years old) at the Dental Hospital of the Faculty of Dentistry, Universitas Indonesia who were ready to start orthodontic treatment. Additionally, they had good oral health and were free of any oral diseases. Periodontal diseases and oral health status were determined by two registered periodontists. The study protocols were approved by the Ethics Committee of the Faculty of Dentistry, Universitas Indonesia (ethical reference numbers: 123/Ethical Approval/FKGUI/XII/2024), and all participants gave written informed consent prior to participation in the study in accordance with the requirements of the Ethics Committee. The study only included participants who were non-smokers, had no periodontal disease (gingivitis and chronic periodontitis) or systemic diseases, and had no history of antibiotic use within the previous three months. Participants with systemic or local conditions that impact bone metabolism and poor dental hygiene, measured by the simplified Oral Hygiene Index (OHIs) [29], were not allowed to participate in the study. Patients in this study were classified as having adequate oral hygiene if their OHIs levels were less than 1.2 and as having poor oral hygiene if their OHIs levels were more than 3.

### 2.2. Collection of Saliva, Dental Gingival Crevicular Fluid, and Peri-Mini Implant Crevicular Fluid Samples

**Figure 1:**
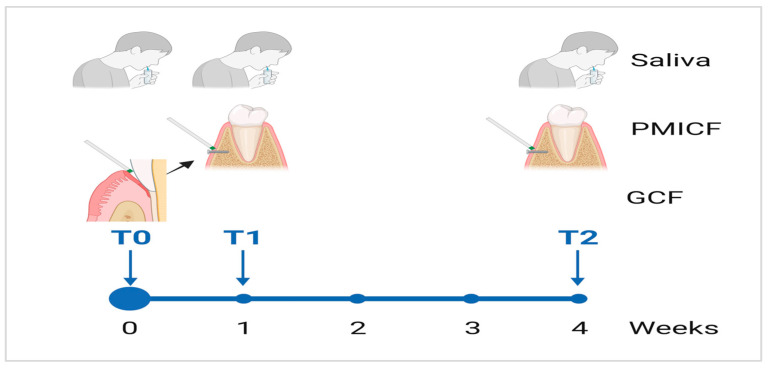
This timeline illustrates the use of a longitudinal study design for oral fluid collection. A thorough baseline collection is performed at T0, a targeted collection is carried out at T1, and a final comprehensive sample is collected at T2 in order to assess and analyze changes in the microbial composition over a four-week period. BioRender was used to generate this illustration (https://biorender.com/).

Saliva and dental gingival crevicular fluids (GCF) samples were obtained at baseline (T0) to assess changes in oral microbiota after mini-implant (MI) installation. Saliva, GCF, and peri mini-implant crevicular fluids (PMICF) samples were taken one week (T1) and four weeks (T2) after MI placement (Figure 1). The saliva sample at T0 provides a comprehensive oral microbiome profile, whereas GCF at T0 serves as an initial evaluation of the native microflora.

Participants provided 2 mL of unstimulated saliva by spitting into a sterile 15 mL screw cupped centrifuge tube. For GCF and PMICF collection, participants first rinsed with sterile water. Cheek retractors were placed, and the gingival crevicular area or implant site was isolated and dried with cotton rolls. A sterile paper point was then inserted into the sulcus between the transmucosal neck of the implant and the attached gingiva, where it remained for one minute. Despite having similar functional traits, GCF and PMICF may have different metabolic profiles because of variations in tissue composition and cellular activity [30]. In this study, the same procedure was used to collect GCF at T0. After collection, paper points were placed in 500 µL microcentrifuge tubes containing phosphate-buffered saline (PBS). All samples were immediately transported to the lab for centrifugation at 2000 rpm to separate GCF/PMICF from paper point. Samples were kept at −80 °C until DNA extraction.

### 2.3. DNA Extraction, Sequencing and Real Time-PCR

Using the Monarch^®^ Genomic DNA purification kit, NEB #T3010S/L (New England Biolabs, Frankfurt am Main, Germany), the samples were extracted in accordance with the company’s directions. A Qubit 2.0 Fluorometer (Invitrogen, Carlsberg, CA, USA) was used to measure the concentration of the extracted DNA. Following PCR amplification, PCR products from each sample were purified and quantified using the Qubit. In the present study, samples from the same site were pooled at each time point to produce a more representative average of the microbial community, considering the greater similarity of microbiomes from the same location across different individuals [31]. Thus, the genomic DNA of each time point from subjects was mixed in equimolar quantities to create a pool of three group libraries for nanopore sequencing. Subsequently, the 16S Barcoding Kit 24 V14 (SQK-RAB204, Oxford Nanopore Technologies, Oxford, UK) was used to construct the nanopore amplicon library in accordance with the manufacturer’s instructions. Primers 27F and 1492R are included in the kit to amplify the whole 16S rRNA gene. There were 100 ng of starting DNA in each barcoding kit. The MinION (Oxford Nanopore Technologies, Oxford, UK) with a MinION flow cell (R10.4.1) was used for 20 h of sequencing. Real-time PCR was used to assess and compare the expression levels of C-reactive protein/CRP, IL-6 (host response-associated indicators), *narG*, and *napA* genes (NRB-related activities), while GAPDH was used as the internal control (housekeeping gene) to normalize the transcription levels [28]. The interaction between Saccharibacteria and interleukins was investigated using a PCR technique and previously published primers [17,32].

### 2.4. Data Processing and Analysis

Using the Dorado 7.6.8 basecaller, the raw sequencing data was base-called. To maintain high-quality reads, the reads were trimmed and filtered based on a minimum quality score (q-score ≥ 8). The nextflow run wf-metagenomics pipeline was used to process the resulting pass reads for taxonomy classification using the Kraken2 database (ncbi_16s_18s_28s_ITS). Using Kraken2 and the designated database, taxonomic assignment was performed at the phylum to species level, with minimum and maximum read lengths for classification set at 200–1500 bp. Operational Taxonomic Units (OTUs) were created from the underlying taxonomy data. Using qiime2 vsearch clustering, Saccharibacteria OTUs were identified, and qiime2 feature-classifier classi-fy-sklearn was utilized to assign taxonomy using th Sil-va-138-99 classifier (https://docs.qiime2.org/2024.10/data-resources/ 22 February 2025). Alpha and beta diversity studies were performed using the vegan, ggplot2, and dplyr packages in R (version 4.3.2). The Simpson and Shannon indices were utilized to measure alpha diversity, which measures the variety of bacterial species present in each saliva or PMICF/GCF sample. Beta diversity, which compares the differences in bacterial composition between saliva and PMICF/GCF or over time, was computed using Bray–Curtis dissimilarity, and differences were assessed through PERMANOVA. Additional studies, including Venn diagrams, heatmaps, and OTU analysis, used R. In R, microbial networks were constructed using the igraph and ggraph packages (version 4.3.2) for network generation and visualization (Fruchterman-Reingold layout), the tidyverse package for data processing, and Pearson correlation analysis (|r| > 0.1). For qPCR results, GraphPad PRISM 9.4 was applied for statistical analysis. The statistical significance level was set at *p* < 0.05. One-way ANOVA with Tukey’s HSD was used for intragroup comparisons, while two-way ANOVA was used for intergroup comparisons.

## 3. Results

Clinical monitoring verified that appropriate oral hygiene was practiced throughout the course of the study (T1 and T2). There was no spontaneous bleeding, change in the color (erythema) or swelling of the keratinized gingiva surrounding the MI. Given the fact that some of our participants experienced mild discomfort during the research period, we expected that the biology process would involve an inflammatory response, local and systemic, during orthodontic treatment [33,34]. Consequently, it may affect oral habitat.

### 3.1. Diversity and Evolution of Microbes over Time

**Figure 2:**
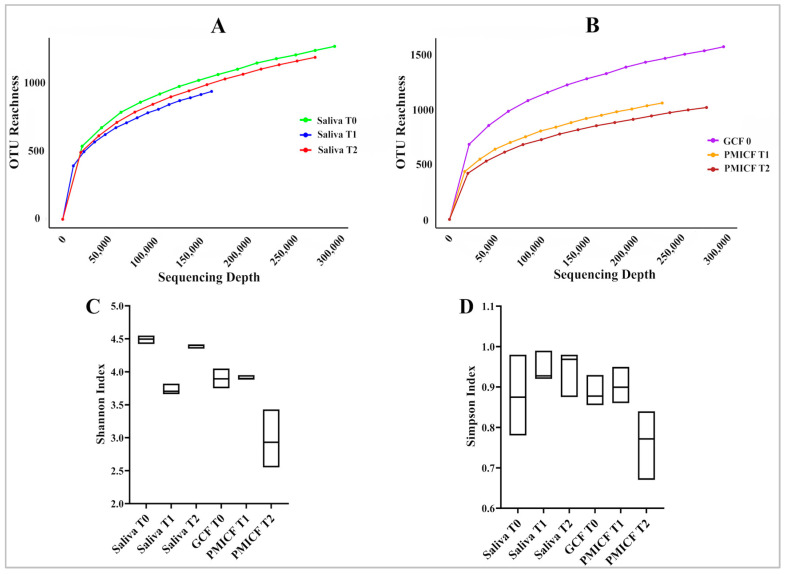
Analysis of microbial diversity and richness. A rarefaction curve illustrating the relationship between sequencing depth and observed OTUs in saliva samples (**A**) and the relationship between sequencing depth and observed OTUs in gingival crevicular fluid (GCF) samples (**B**) are shown in the upper panel. The lower panel shows the alpha diversity, indicating Shannon (**C**) and Simpson (**D**) indices along with box plots of the saliva and GCF sample values at baseline (T0), time point 1 (T1), and time point 2 (T2).

Sufficient sequencing depth for saliva, GCF, and PMICF samples was established by rarefaction curves (Figure 2A,B). Results of alpha diversity showed that the bacterial richness (Shannon index) of saliva was higher at baseline (T0) than at T1, and its evenness (Simpson index) remained constant across time (T0, T1, and T2) (Figure 2C,D). Additionally, compared to T0, which was only based on GCF, and T1, which was measured at PMICF, there was a clear correlation between increases in PMICF and declines in the Shannon Index (diversity) and Simpson Index (evenness) at T2.

It appears fewer species were dominating the microbial ecology surrounding the PMICF. The findings imply that throughout the study period, the core microbiota was continuously found in the surrounding mini-implant and in the healthy gingival crevicular niche. However, after the extended period of four weeks (T2), the microbial composition surrounding the mini-implant had changed significantly.

**Figure 3:**
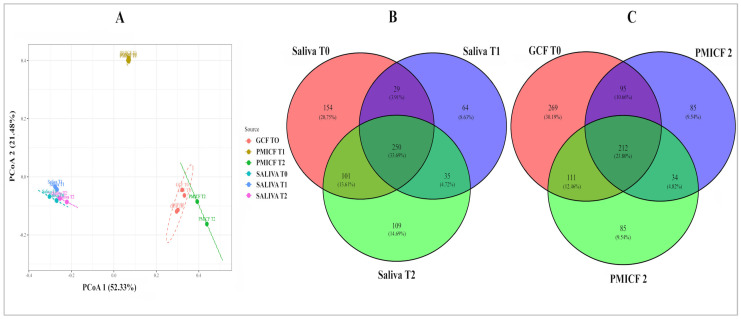
Illustration of the differences in the microbial communities and shared taxa among the tested oral samples. (**A**) Principal coordinate analysis (PCoA) based on beta diversity metrics reveals the distinct clustering of microbial communities from saliva, GCF (T0), and PMICF (T1 and T2). Each time point represents a sample, and special separation reflects differences in microbial composition over time and between sites. The percentage of variation explained by each axis is shown. Venn diagrams (**B**,**C**) that display the quantity of microbial and shared taxa found in saliva and GCF/PMICF, respectively. Taxa found in different sample types are indicated by overlapping areas, which might indicate a core or transitional microbiota between oral conditions.

The distinct microbial habitats indicated by the Shannon and Simpson index values, which clearly separate saliva from GCF/PMICF, were further validated by the beta diversity, using principal coordinate analysis (PCoA) (Figure 3A). Despite a reduction in alpha diversity at T2, beta diversity analysis shows that the microbial community composition of PMICF at T2 remains similar to that of GCF at T0 and PMICF at T1.

Lastly, core microbiome members were identified using Venn diagrams, with 30.9% of GCF-PMICF OTUs and 46.8% of salivary OTUs consistently identified at all three points (T0, T1, and T2). Figure 3B,C demonstrate that while OTUs were more prevalent in saliva at baseline (T0), PMICF samples at T1 and T2 had fewer OTUs in total when compared to GCF assessed at T0. This was consistent with the decrease in Shannon index that we observed.

### 3.2. Overall Taxonomic Alteration and Microbial Composition at Phylum and Genus Level

**Figure 4:**
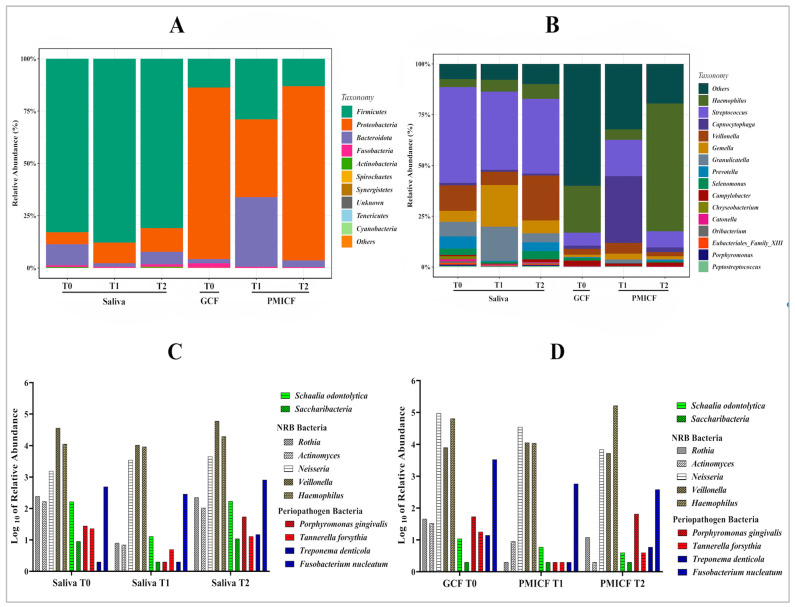
Bacterial composition and key selected taxa in saliva and peri-implant crevicular fluid (PMICF). (**A**) Phylum-level distribution of bacteria in saliva and GCF/PIMCF at baseline (T0) and after MI placement (T1 and T2). The graph shows the relative abundance (%) of various bacterial phyla, including *Firmicutes*, *Actinobacteria*, *Bacteroidota*, and *Proteobacteria, and Fusobacteria*, across the different sample types and time points. (**B**) Genus-level distribution of bacteria in saliva and GCF/PMICF samples. The graph shows the relative abundance (%) of key genera, including nitrate-reducing bacteria (the figure focuses on *Haemophilus* and *Veillonella*, while *Actinomyces*, *Rothia*, and *Neisseria*, are not individually represented), and periodontopathogen bacteria (*P. gingivalis*, *T. forsythia*, *T. denticola*, and *F. nucleatum*). (**C**) Relative abundance of selected bacteria in saliva samples at baseline (T0) and after mini-implant placement (T1 and T2). (**D**) Relative abundance of selected bacteria in GCF (T0) and PMICF (T1, T2) samples.

The classification of microorganisms revealed 23 phyla, 49 classes, 117 orders, 301 families, and 1151 genera. *Firmicutes*, *Bacteroidota*, *Fusobacteria*, and *Proteobacteria* dominated saliva (Figure 4A). Dynamic changes were noted at several taxonomic levels: *Firmicutes* continued to dominate saliva, though with a slight decline over time. *Proteobacteria* predominated in GCF at baseline, but percentage of the bacteria in the peri-implant gingival site decreased somewhat after a week and rose to the same level at the T2 time point.

Figure 4B displays the relative abundance of the top 15 genera across all samples and time points. The relative abundance of the dominating genus, especially *Streptococcus*, in saliva generally stayed rather stable, but *Veillonella*’s abundance rose after four weeks (T2) after significantly declining at 1 week (T1). After four weeks, the most noticeable effect was shown in *Haemophilus* at the expense of *Proteobacteria*. Furthermore, compared to the baseline number in GCF, the proportion 0.0737% of *Porphyromonas* and 32.27% *Capnocytophaga* in PMICF increased after one week before declining at four weeks.

### 3.3. Saccharibacteria, Nitrate-Reducing Bacteria, and Periodontopathogens

Figure 4C,D illustrate how the relative abundance of Saccharibacteria and *S. odontolytica* appears to decrease with time when comparing PMICF to saliva. This could suggest that inflammation and mini-implant placement have impacted on their presence. The nitrate-reducing bacteria (*Rothia, Actinomyces*, *Neisseria*, *Veillonella*, and *Haemophilus*) were comparatively abundant and stable in saliva (T0, T1, and T2). The prevalence of these bacteria remains high generally, despite a few slight differences.

When comparing GCF (T0) to PMICF (T1, T2), NRB were also dominant in PMICF at T1 and T2, with no discernible decrease over time. Their stability raises the possibility that they play a part in preserving a healthy microbial ecology and preventing the overabundance of periodontopathogens. For periodontopathogens (*P. gingivalis, Tannerella forsythia, Treponema denticola*, and *Fusobacterium nucleatum*), low concentrations were found in saliva (T0, T1, T2), although *P. gingivalis* was found to have slightly increased at T2. In GCF (T0) vs. PMICF (T1, T2), the number of periodontopathogens increased in PMICF following mini-implant insertion (T1 and T2). Despite this increase, NRB still appeared to outcompete periodontopathogens in relative abundance.

### 3.4. Microbial Interaction and Functional Relationships

**Figure 5:**
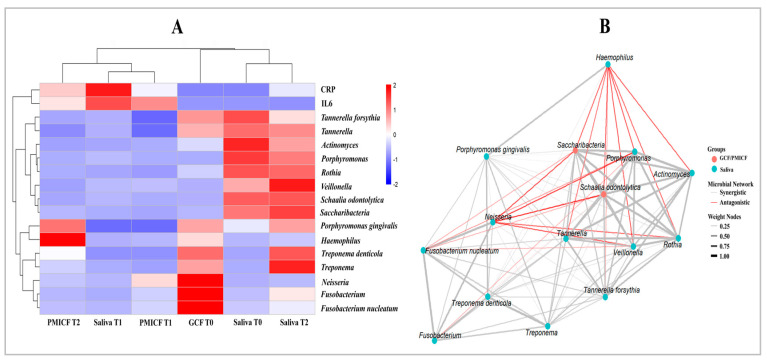
Heatmap correlation and co-occurrence study of specific bacterial groupings and inflammatory indicators. (**A**) The heatmap displays the relationships between a few bacterial taxa, including Saccharibacteria, its host (*Schaalia odontolytica*), nitrate-reducing bacteria (NRB), and periodontopathogens, and host inflammatory markers (CRP and IL-6). While NRB and Saccharibacteria and periodontopathogens exhibited negative correlations with IL-6, NRB might have an anti-inflammatory function in regulating inflammation. (**B**) Co-occurrence analysis suggesting potential relationship between microorganisms. A dependent connection was suggested by the positive co-occurrence of Saccharibacteria and *S. odontolytica*. The co-occurrence of periodontopathogens in dysbiotic biofilms supports their synergism, but NRB exhibited negative connections with periodontopathogens, suggesting antagonistic relationships.

As can be seen from the heat map (Figure 5A), which depicts changes in microbial composition over time, Saccharibacteria and *S. odontolytica* declined in GCF, indicating that ecological modifications had an impact on their survival. The biological relationship between these bacteria was strengthened as they developed a separate sub-cluster. *Fusobacterium nucleatum* and other periodontopathogens rose in GCF while NRB decreased. By grouping NRB together, clustering analysis indicated that they share a metabolic function in nitrate reduction. Furthermore, the co-occurrence network (Figure 5B) revealed positive correlations between the periodontopathogens (*P. gingivalis, T. denticola, T. forsythia*, and *F. nucleatum*), indicating cooperative biofilm formation and microbial interactions. Saccharibacteria and periodontopathogens exhibited antagonistic associations, indicating a competitive dynamic, whereas *F. nucleatum* showed significant interactions with both red complex bacteria and Saccharibacteria, suggesting a potential role as a bridge species in dysbiotic changes [35].

### 3.5. Transcription Patterns of CRP and IL-6

**Figure 6:**
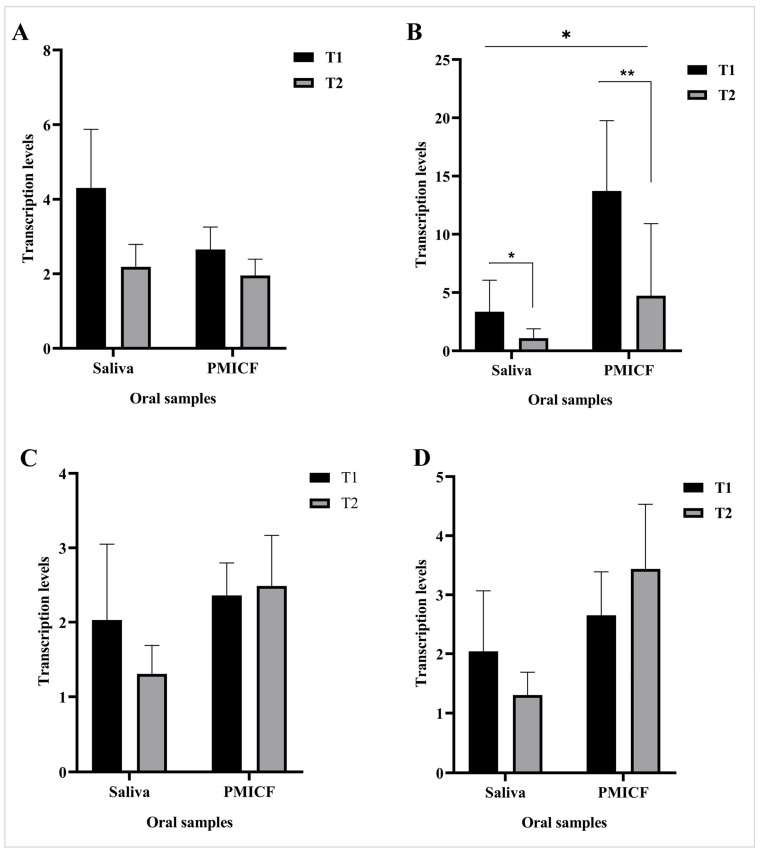
Comparative analysis of the transcription of CRP (**A**) and IL-6 (**B**) (host inflammatory response) and *narG* (**C**) and *napA* (**D**) (nitrate-reducing bacterial activity) and in saliva and PMICF at time points T0, T1, and T2. For each sample type, T0 is the within-subject baseline comparator. In both saliva and PMICF, IL-6 levels were elevated at T1 and decreased by T2, indicating a gradual decrease in inflammation. CRP levels remained mostly stable. Differing sample types had different levels of *narG* and *napA* transcription, but saliva generally had greater levels, suggesting a better nitrate-reducing activity than PMICF. * *p* < 0.05, ** *p* < 0.01.

As shown in Figure 6 in comparison with T0, both mRNA transcription levels CRP and IL-6 rose following T1 and T2, suggesting that following mini-implant implantation, inflammation escalated and continued at both time points. Moreover, at each time point (T1 and T2), the transcription levels of CRP in saliva and PMICF were similar. In contrast, saliva and PMICF had significantly different levels of IL-6 expression, with PMICF expressing the inflammatory signal at higher levels than saliva (*p* < 0.05). However, in both saliva and PMICF, IL-6 levels dramatically dropped from T1 to T2.

## 4. Discussion

This study investigated how the oral microbiome changes after mini-implant implantation, specifically in saliva and a new location called the peri mini-implant crevicular fluid (PMICF) that forms around the implant [36]. Clinical monitoring over the study period (T1 and T2) showed no evidence of inflammation, good systemic condition, and good oral hygiene (OHIs), despite some individuals experiencing slight discomfort when the micro implant was implanted. For this reason, we evaluated the possibility of whether microbial shifts may serve as early microbial markers of peri-implant mucosal alterations prior to the appearance of visible inflammation. Therefore, to gain a better understanding of the relationship between MI-orthodontic devices and oral health, we concentrated on groups of oral bacteria; periodontal pathogens, Saccharibacteria, and NRB. Our data indicate that the observed microbial patterns are not primarily caused by poor oral hygiene and remove any confounding variables associated with systemic diseases. Consequently, tissue injury may not always be the outcome of changes in the microbial community’s structure caused by host or environmental stresses [37,38,39].

### 4.1. Microbial Diversity and Stability Post-Mini Implant Placement

Overall, during a one-month period, this study showed dynamic changes in the oral microbiota of MI patients. The finding supports a previous study that found bacteria can develop around mini-implants in less than a day [40]. In this study, saliva and peri-implant crevicular fluids (PMICF) microbiomes were compared as an overall view of the oral microbiome’s reaction to the mini-implant can be obtained from saliva samples. Saliva samples show shifts in the whole-mouth oral microbiome and offer a similar background to the localized microbial changes seen in PMICF. This can show whether the implant has systemic effects beyond its immediate location. In a prior study, patients with cardiovascular illness showed decreased richness and evenness in subgingival microbiota, which would indicate a higher level of microbial homogeneity [41]. Since we concentrated on the microbiome surrounding the mini-implant, the current investigation and that study cannot be directly compared; yet, we speculated that our patient’s salivary microbiota, which is free of systemic disorder, could be resilient and adaptive. Thus, the microbial alteration in saliva and PMICF were first validated using rarefaction curve analysis, which demonstrated adequate sequencing depth.

With respect to both alpha and beta-diversity, the oral microbiome profile of the saliva sample was considerably different from PMICF, suggesting that they are distinct microbial environments. Alpha diversity analysis showed that while salivary bacterial evenness (Simpson index, close to 1) remained stable throughout time, bacterial richness (Shannon index) in saliva reached baseline (T0) and declined at T1. The greater diversity of bacteria in saliva from T1 to T2 indicated that the salivary microbiome had been further restored to its original state. This indicates that the initial disruption induced by the mini-implant implantation was only transient. A more stable and resilient community is further suggested by the PCoA plot, which also demonstrates that the recovery was consistent with the relatively closer grouping of saliva samples. Given that the primary source of salivary microorganisms is the biofilm that is shed on the oral tissue’s surface [42], the presence of mini-implants may have an impact on it.

We argue that the overall balance of bacterial species observed in saliva is a reflection of local ecological variables associated with the mini-implant. In contrast, PMICF exhibited a decline in both richness and evenness at T2 (compared to T1 and GCF at T0). This implies that the mini-implant reduces microbial diversity and leads to a shift in the PMICF community toward a population that is less evenly distributed. However, comparisons between T0 (GCF) and T1/T2 (PMICF) might not correctly represent temporal changes, but rather site transformation that may affect the microbial profile. Additionally, the study lacks biological replicates due to sample pooling. As a result, statistical findings should be considered solely for informative purposes. This restriction renders it unattainable for us to attribute observed microbial shifts to a chronological trend exclusively; instead, they should be described as differences between the ecosystems before and after implants.

Additionally, beta diversity revealed that a higher dispersion of PMICF samples in the PCoA analysis indicates a shift towards a less diverse and more variable population. This corresponded with PMICF decreasing Simpson and Shannon diversity over time. Therefore, our results demonstrated that after a month, the peri-implant environment shifted toward a more dysbiotic state, although there were no overt signs of inflammation in the participants who received mini-implants. This result is particularly relevant to the increase of *P. gingivalis* and *F. nucleatum*, the pathobionts of periodontal disease [43,44], and the gradual decline in several nitrate-reducing bacteria (NRB), particularly *Actinomyces* and *Rothia* that we found at T2 in PMICF. Both *Actinomyces* and *Rothia* are predominant in the healthy oral cavity compared with periodontitis [45]

Overall beta diversity in PMICF remained similar at T1 and T2, suggesting that the microbial community surrounding the mini-implant stabilized after an initial period of disruption. The Venn diagram data, which indicate a drop in OTUs in PMICF at T1 and T2 compared to GCF at T0, reinforce this finding by showing a decreased level of microbial diversity in the peri-implant environment. However, the higher proportion of consistent OTUs (46.8%) in saliva as compared to GCF/PMICF (30.9%) indicates that the salivary microbiome is more stable over time. Likewise, when other taxonomic classifications (phylum, genus) were taken into account, a shift in consistency was observed in the results. We noticed that overall taxonomic profiling showed a slight shift over time across various classification levels in both saliva and GCF/PMICF data.

Consistent with a previous study, the dominant phyla at the phylum level were *Firmicutes*, *Proteobacteria*, *Bacteroidota*, *Fusobacteria*, and *Actinobacteria* [24,46]. Interestingly, in line with an earlier study [47], we found that Saccharibacteria were detected at a low abundance. Saccharibacteria have been demonstrated to parasitize other oral bacteria, which can impact the structure, hierarchy, and functionality of the microbiome in certain contexts [47]. In our study, the presence of Saccharibacteria correlated positively with IL-6 levels and shifts in the biofilm composition in the mucosa surrounding the MI. Furthermore, the consistent fluctuation indicated over the duration of the four weeks suggests the possibility that Saccharibacteria function as a relatively stable component of the microbial network rather than as a potent antagonist or promoter of dysbiosis. Although the current evidence is unable to demonstrate causation, this apparent stability may be correlated to the maintaining of microbial homeostasis; thus, this relationship should be considered as an association. To ascertain whether Saccharibacteria directly affect the abundance of other bacterial species or if they instead co-vary with other ecological changes in the peri-implant environment, more research is required with larger sample sizes and suitable control groups.

Moreover, we observed that in saliva, *Firmicutes* maintained a relative high abundance across all time points, although a slight decrease was noted from T0 to T2. In contrast, PIMCF samples exhibited a marked reduction in *Actinobacteria* from T0 (observed in GCF) to T2 (observed in PMICF), accompanied by relative rise in *Proteobacteria* following MI placement. According to a previous study, *Actinobacteria* are more commonly associated with health [22]. Our finding that *Actinobacteria*, a phylum that includes *S. odontolytica*, significantly decreased from T0 to T2 raises the possibility of an early microbial homeostasis disturbance in the peri-implant environment.

At the genus level, the data highlighted a shift in key genera, including Saccharibacteria-*Schaalia* (*Actinomyces*) complex. Particularly, *S. odontolytica* was consistently detected at T0 but declined significantly at T1 and T2 in PMCF. Other genera, such as nitrate-reducing bacteria (*Neisseria* and *Rothia*) also displayed variations in relative abundance. *Haemophilus* grew at the expense of other *Proteobacteria*, while *Veillonella* abundance temporarily decreased at T1 but increased at T2. Our research suggests that the formation of plaque is a risk associated with placing mini-implants [48]. The presence of mini-implants affects not only the targeted pathogens and symbionts but also the entire taxonomic structure of the oral microbiome.

It has been shown that pathobionts belonging to the *Actinomyces* genera, including *S. odontolytica* (Saccaharibacteria host), which is a member of *Actinobacteria*, contribute to periodontal inflammation [23,49]. Our results suggest that Saccharibacteria/host bacterial interactions that persist in the biofilm surrounding mini-implant after it has been placed may lead to resistance to the inflammation induced by the host bacteria (*S. odontolytica*), as it belongs to nitrate-reducing bacteria. Likewise, our research confirms previous findings that *Saccharibacteria* are present in a variety of oral niches, in both healthy and pathological conditions [20,50,51].

### 4.2. Stability of Saccharibacteria, Nitrate-Reducing Bacteria, and Periodontopathogens

This study specifically aimed to determine how the pattern of interaction among Saccharibacteria, nitrate-reducing bacteria (NRB), and periodontopathogens contributes to the formation of homeostatic biofilm following mini-implant orthodontics placement. Our data indicate that periodontopathogens (*P. gingivalis*, *T. denticola*, *T. forsythia,* and *F. nucleatum*) were detected in saliva at low levels but elevated in PMICF at T1 and T2, indicating a change to a more dysbiotic environment. It is important to note that these periodontopathogens are not always reliably represented in saliva, as they tend to be subgingival specialists and may not be shed significantly into whole-mouth samples, particularly in early or localized disease [52,53]. Supporting this, mouth rinsing-based sampling detects *P. gingivalis* and other pathogens at significantly lower frequencies than subgingival plaque sampling, underscoring its limited sensitivity to subgingival colonization [54]. Conversely, absence from saliva does not confirm absence from specific peri-implant sites, highlighting the importance of combining whole-mouth and site-specific sampling to accurately capture both generalized and localized microbial shifts. This complementary sampling approach provides a more comprehensive understanding of microbial changes during mini-implant placement and helps distinguish localized effects from broader oral microbiome trends.

In contrast, both saliva or PMICF consistently contained nitrate-reducing bacteria (NRB), such as *Rothia*, *Actinomyces*, *Neisseria*, *Veillonella*, and *Haemophilus*. All of them are prevalent in healthy individuals’ oral microbiomes [24]. Similarly, in either saliva or PMICF, Saccharibacteria and *S. odontolytica* were consistently found in relative lower abundances at all time points compared with periodontopathogen and the NRB.

The trends were confirmed by the heat map and co-occurrence network, showing clear interaction dynamics and clustering patterns. The positive correlation and coherence clustering of NRB (*Neisseria*, *Haemophilus*, and *Veillonella*) suggested possible metabolic collaboration and oral health maintenance [26]. However, our data showed that Saccharibacteria and its host (*S. odontolytica*) showed antagonistic associations with key periodontopathogens (*P. gingivalis*, *T. denticola*, and *F. nucleatum*) and clustered separately. Despite the fact that the bacteria have been linked in numerous studies to the onset of periodontal disease [55,56,57], our data might suggest competitive exclusion. Thus, our results validated the idea that Saccharibacteria and NRB might function as ecological stabilizers, preventing the growth of dysbiotic communities [47]. Specifically, *F. nucleatum* seemed to be at the core of the network, interacting with both red complex and NRB species. This supports its proposed function as a microbial bridge during the formation of biofilms and the onset of inflammation [22,35,58].

Oral microbiome study has extensively documented niche-driven ecological alterations [59], which in our study are reflected in the different microbial profiles between saliva and GCF/PMICF. As we observed in the current study, saliva retained a greater diversity of microorganisms, with nitrate reducer/NRB (*Neisseria*, *Veillonella*, and *Haemophilus*) continuing to be the predominant species. This finding supports the emerging concept that NRB contribute to microbial homeostasis by counteracting dysbiotic shift driven by keystone periodontopathogens [13,60]. However, the decrease in Saccharibacteria, *S. odontolytica*, and *Actinomyces* in PMICF could be an indicator of an ecological pressure promoting anaerobic-associated periodontopathogens. Hence, the presence of *S. odontolytica*, a nitrate-reducing *Actinomyces* spp. that hosts Saccharibacteria, further suggest a complex interplay between commensal and pathogenic bacteria in peri-mini-implant environments. Additionally, the consistent fluctuation over the course of the four weeks suggests that Saccharibacteria may not be a strong antagonist or promoter of dysbiosis, but rather an inert component of the microbial network. Consequently, it may support the homeostatic stability of microorganisms. To understand more about how the Saccahribacteria and *S. odontolytica* combination alters the microbiota surrounding peri mini-implants, additional study is required.

While Saccharibacteria may prevent dysbiosis by preventing its host role as a pathobiont [23], dysbiosis itself involves inflammation [61]. We observed that IL-6 levels significantly declined from T1 to T2 in both saliva and PMICF. Over that period, PMICF’s IL-6 levels dropped, indicating that as the healing process for MI installation progresses, implant inflammation will steadily diminish. Alongside this decline, the microbiome shifted, with a relative decrease in periodontopathogens and an increase in nitrate-reducing bacteria (NRB). In order to facilitate tissue repair, the elevated level of NRB may help reduce inflammatory stimulation while supporting a more balanced biofilm. Although this trend points to a possible connection between alterations to the microbial community and the host’s inflammatory response, the limitations of the present study make it difficult to provide evidence of causation, therefore these findings should be considered with caution.

Nevertheless, CRP expression showed a similar decreasing tendency, indicating the positive correlation between both cytokines during inflammation [62], yet there was no statistically significant difference between T1 and T2. This discrepancy may result from the distinct roles that IL-6 and CRP play within the inflammatory cascade, with IL-6 acting as a rapid responder and CRP typically reflecting longer-term systemic adjustments [32]. Importantly, during the research period, our patients maintained good oral hygiene and good general health, which would have allowed us to explain why the inflammatory response was mild and self-limiting.

Alongside this loss, the microbiome underwent a transformation characterized by a rise in nitrate-reducing bacteria (NRB) and a relative drop in periodontopathogens. The increased presence of NRB may assist in minimizing inflammatory stimulation and promote a more balanced biofilm to aid in tissue repair. The constraints of the current study make it hard to establish causation, thus these results should be interpreted cautiously even though this pattern suggests a potential link between alterations in the microbial community and the host’s inflammatory response.

Conversely, between T1 and T2, the transcription levels of two important genes involved in nitrate reduction (*napA* and *narG*) [63] were mostly unchanged. As we pointed out in PMICF, this could result in a lower IL-6 level [64]. Despite this, the consistent long-term abundance of NRB and the stability of Saccharibacteria suggest a counterbalancing effect that may help regulate inflammation-immune homeostasis [65] and microbial dysbiosis.

The limitations of this investigation are inherent. First, this study’s sample size was limited. The limited sample size may affect the generalizability of the results. A larger group would yield more statistically significant results. Second, the absence of a separate control group of patients who did not undergo mini-implant placement is one of the study’s limitations. Instead, PMICF recorded at T1 and T2 were compared with baseline GCF (T0) and saliva samples as within-subject reference points. This approach does not provide the same level of external comparison as a true no-procedure control group, yet it allowed us to observe site-specific and temporal changes within the same individuals. Future studies need to include similar controls that can differentiate between changes caused by the procedure and those arising from the typical temporal variation of the oral flora. Third, the study assessed microbial alterations for a month following the placement of the mini-implant. To determine whether these changes persist, stabilize, or result in clinical problems like peri-implant inflammation, further follow-up is required. Additionally, taxonomic profiling was the main method used in the study. Deeper understanding of the functional effects of periodontopathogen enrichment and nitrate-reducing bacteria depletion may be feasible through metatranscriptomic or metabolomic approaches.

## 5. Conclusions

This study reveals a microbiological transition following mini-implant placement, which is illustrated by a decline in Saccharibacteria, enrichment of periodontopathogens, and depletion of NRB. The red complex (*T. denticola*, *T. forshytia*, and *P. gingivalis*) and *F. nucleatum* have strong associations, suggesting that the latter may play an important role in early dysbiotic alterations. These findings could also suggest early biomarkers for oral microbial health and future strategies for preserving the microbial balance around orthodontic mini-implants. However, the use of pooled data is an important constraint, and further study using individual biological duplicates is necessary to confirm these findings and draw statistical inferences.

## Data Availability

The original contributions presented in the study are included in the article. Raw data saliva, GCF, and PMICF microbiome using 16s barcoding kit 24 V14, ONT (Oxford Nanopore Technology) were available in DOI: 10.6084/m9.figshare.29257364. Data are available under the terms of the Creative Commons Zero “No rights reserved” data waiver (CC0 1.0 Public domain dedication).

## Abbreviations

GCF	Gingival Crevicular
PMICF	Peri mini-Implant Crevicular Fluids
NRB	Nitrate-Reducing Bacteria
OHIs	Oral Hygiene Index simplified
OUT	Operational Taxonomic Unit
CRP	C-reactive protein
IL-6	Interleukin-6
